# Recurrent Laryngeal Nerve Stretching in Tracheal Diverticulum: First Documented Mechanism of Hoarseness and Systematic Review of Literatures

**DOI:** 10.1002/ccr3.71885

**Published:** 2026-02-03

**Authors:** Zahra Sadin, Manouchehr Aghajanzadeh, Mohammadreza Sadin

**Affiliations:** ^1^ Department of Thoracic Surgery Arya Private Hospital Rasht Iran; ^2^ Department of Pulmonology Arya Private Hospital Rasht Iran

**Keywords:** case report, cervical excision, hoarseness, paratracheal air cyst, recurrent laryngeal nerve compression, tracheal diverticulum, vocal cord paresis

## Abstract

Tracheal diverticulum is a rare paratracheal air cyst. It is often asymptomatic. Large symptomatic cases may compress the recurrent laryngeal nerve (RLN) and cause hoarseness. It is a rare presentation, in < 0.2 cases per year in the world. We present a 45‐year‐old woman that had 6 months of chronic cough, dysphagia, odynophagia, dyspnea, hoarseness, hemoptysis, choking, and neck pain. She was not a smoker. Spirometry showed mild obstruction pattern (FEV1 78%). CT with 3D reconstruction revealed a 4 × 5 cm right posterolateral tracheal diverticulum (fifth–seventh rings, 8 mm communication). Bronchoscopy confirmed limited right vocal cord mobility. We did an open cervical excision that preserved the RLN. Histopathology confirmed acquired diverticulum. Hoarseness resolved in 3 weeks; repeat bronchoscopy showed normal vocal cord mobility. 3‐month CT confirmed resolution; 1‐year FEV1 improved. Asymptomatic at 12 months. Systematic review (1998–2025) found 4 prior hoarseness cases (total *n* = 5): 60% female, 80% right posterolateral, mean size 3.0 cm, 80% complete recovery in 3 weeks via open surgery. Largest reported diverticulum is with hoarseness. This is the first case with documentation of RLN stretching and quantitative improvement with spirometry. Multimodal imaging and early open excision with nerve preservation can cause excellent outcomes.

## Key Clinical Message

Tracheal diverticulum causes recurrent laryngeal nerve (RLN) involvement by stretching rather than compression. Multimodal imaging and bronchoscopy help us to make the accurate diagnosis. Early open excision and nerve preservation provide rapid voice recovery. Besides, it also improves objective functional (FEV1 + 10%), by preventing irreversible neural damage.

## 1 Introduction

Paratracheal air cysts (PTACs) are a wide spectrum, which include tracheocele, bronchogenic cysts, lymphoepithelial cysts, and tracheal diverticula. It's prevalence ranges from 0.75% to 8.1%. The incidence of tracheal diverticula (TD) specifically is 2.4%, which is detected by thoracic CT scans [1, 2, 3]. These lesions are defined as air‐filled outpouchings of the tracheal wall and often lined by ciliated columnar epithelium. Interestingly, they occur predominantly on the right posterolateral aspect of the trachea in 97.1% of cases, likely because this area lacks the protective support, while the left side is protected by the aortic arch and esophagus [1, 3].

TD are classified into 2 types: congenital and acquired. Congenital diverticula are smaller and occur more often in males. They are usually located 4–5 cm below the vocal cords with a narrow connection to the trachea. These result from developmental defects in the tracheal cartilage [4, 5]. Acquired diverticula develop at any tracheal level. They are larger and have wider communications. Usually, they chronically increase the intraluminal pressure. It can cause persistent cough or chronic obstructive pulmonary disease combined with tracheal wall weakness. This could describe TD as both a rare cause and consequence of chronic cough. It highlights a bidirectional relationship [5, 6, 7].

They are often asymptomatic and become discovered incidentally during imaging for other conditions [8]. However, they can cause chronic cough, dyspnea, recurrent respiratory infections, hemoptysis, dysphagia, odynophagia, hoarseness, and neck pain. Hoarseness is particularly rare and indicates possible involvement of the RLN. Symptoms begin by increasing its size enough or when complications like infection occur [5, 6].

Multidetector CT with multiplanar reconstruction is the method of choice to evaluate them. Nowadays it becomes the gold standard for visualizing its size, location, relationship to surrounding structures, and communication with the trachea [3, 4]. Recent data shows that for mediastinal cystic lesions, the accuracy of CT is more than 90% [9]. Bronchoscopy by direct visualization can provide complementary information, although the trachea's narrow opening can be difficult to identify [10]. The differential diagnosis includes laryngoceles, pharyngoceles, Zenker's diverticulum, apical lung herniation, and paraseptal blebs [11, 12].

Based on symptom severity, we choose different approaches for asymptomatic patients; the best decision is conservative management with observation and antibiotics [2], but symptomatic cases often require surgical intervention [6]. There is always a probability of some potential complications such as difficult intubation, pneumomediastinum, and respiratory distress [13, 14].

There are only a few detailed case reports that could fully describe a complete clinical picture, diagnostic workup, surgical management, and long‐term outcomes. This is especially true for unusual presentations like hoarseness.

Our case is a 45‐year‐old woman with a broad range of symptoms, such as chronic cough, dysphagia, odynophagia, dyspnea, hoarseness, hemoptysis, choking, and neck pain. We eventually diagnosed a large acquired TD. This report highlights the role of imaging in diagnosis and the excellent outcomes of surgical intervention in symptomatic cases.

## 2 Case Presentation

Our case is a 45‐year‐old woman. Her first complaint was chronic cough, dysphagia, odynophagia, dyspnea, hoarseness, hemoptysis, choking episodes, and neck pain for 6 months. She was a nonsmoker and had a negative past medical history. We found no palpable neck abnormalities in the physical examination, but in auscultation, we detected localized wheezing in the right lung field during deep inspiration. Vital signs were normal, with an oxygen saturation of 98% on room air. Initial CXRAY was unremarkable. Spirometry showed a mild obstructive pattern with FEV1 of 78% predicted and FEV1/FVC ratio of 68%. Routine blood investigations were normal.

**FIGURE 1:**
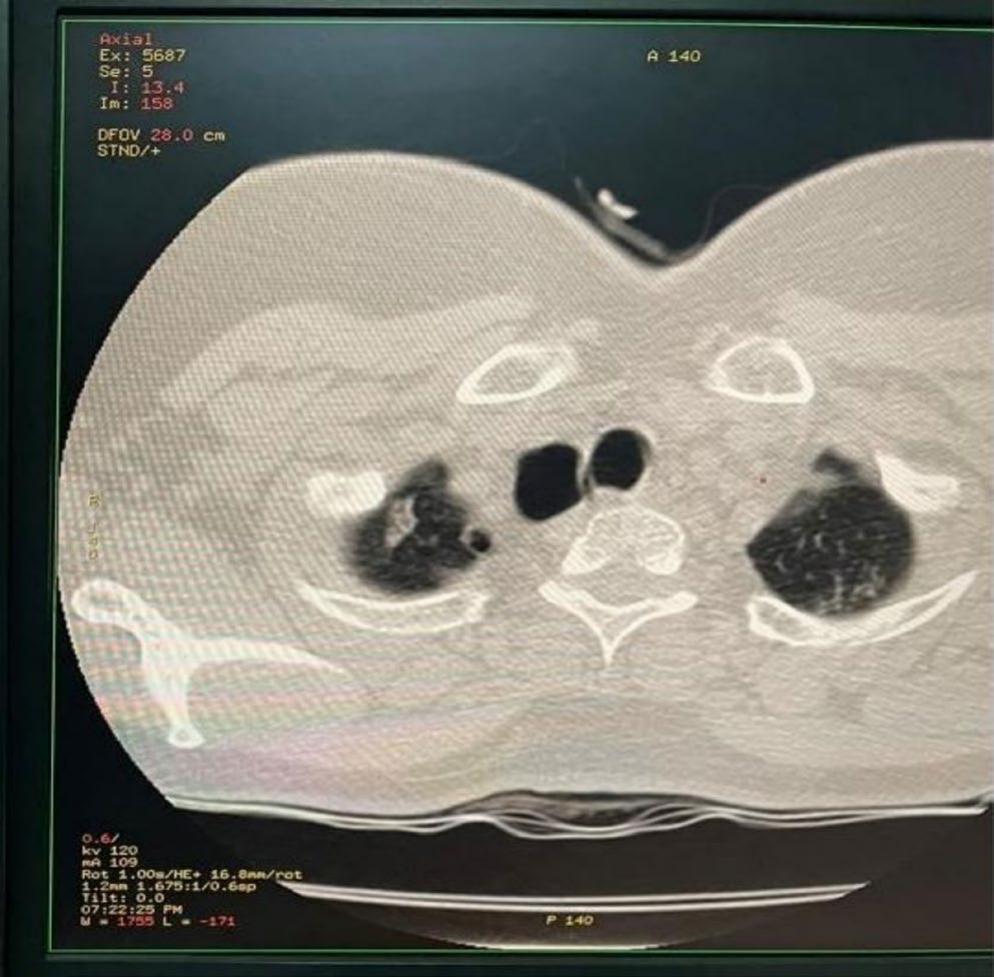
Contrast‐enhanced computed tomography scan of the neck shows a 4 × 5 cm tracheal diverticulum. It is arising from the right posterolateral tracheal wall at the level of the fifth to seventh tracheal rings. The air‐filled outpouching demonstrates clear communication with the tracheal lumen.

**FIGURE 2:**
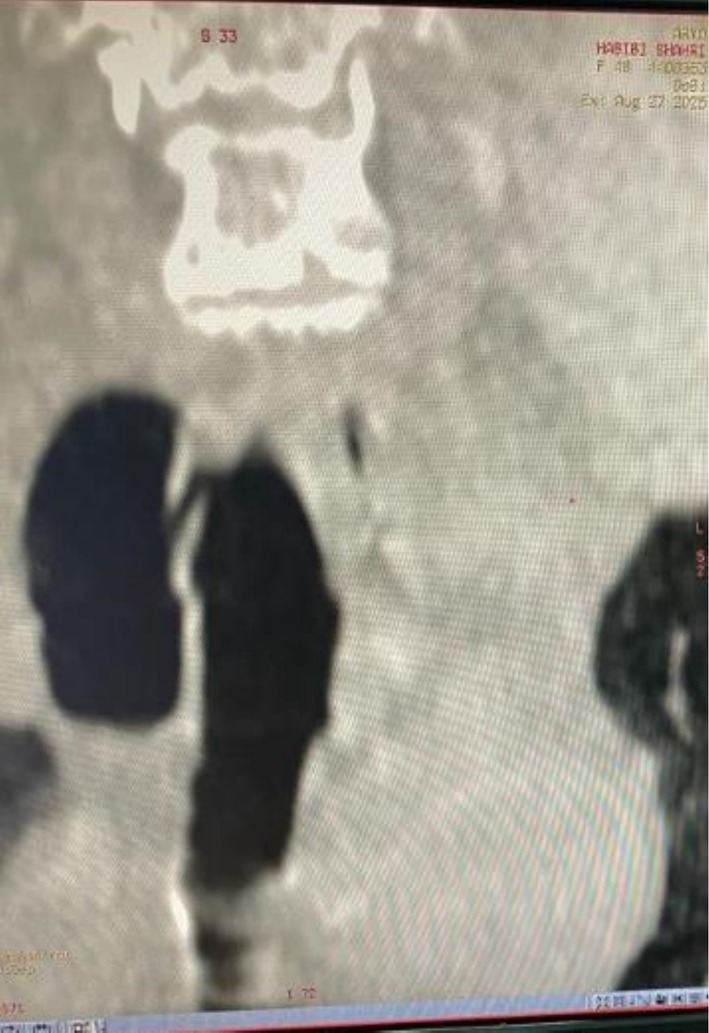
Coronal reformatted CT image demonstrates the tracheal diverticulum in the right posterolateral position. The image shows the cranio‐caudal extent of the lesion and its relationship to surrounding structures.

**FIGURE 3:**
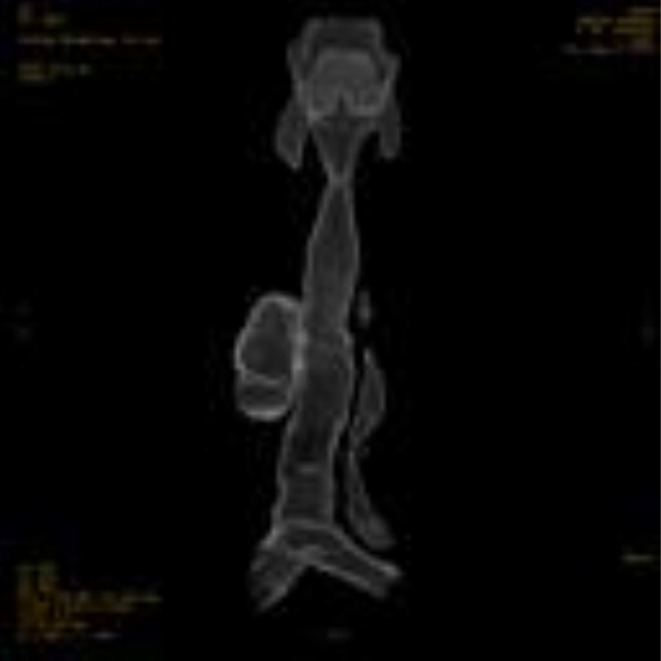
Sagittal reformatted CT image. It shows the posterior location of the 4 × 5 cm diverticulum relative to the tracheal lumen and also its connection at the fifth to seventh tracheal ring level.

**FIGURE 4:**
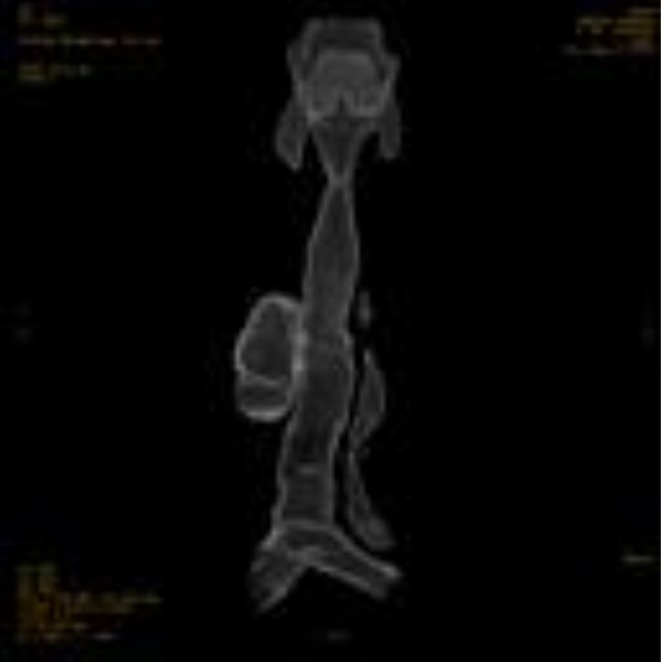
3D CT reconstruction clearly visualizes the tracheal diverticulum and its anatomical relationship to the trachea. This reconstruction was invaluable for surgical planning.

**FIGURE 5:**
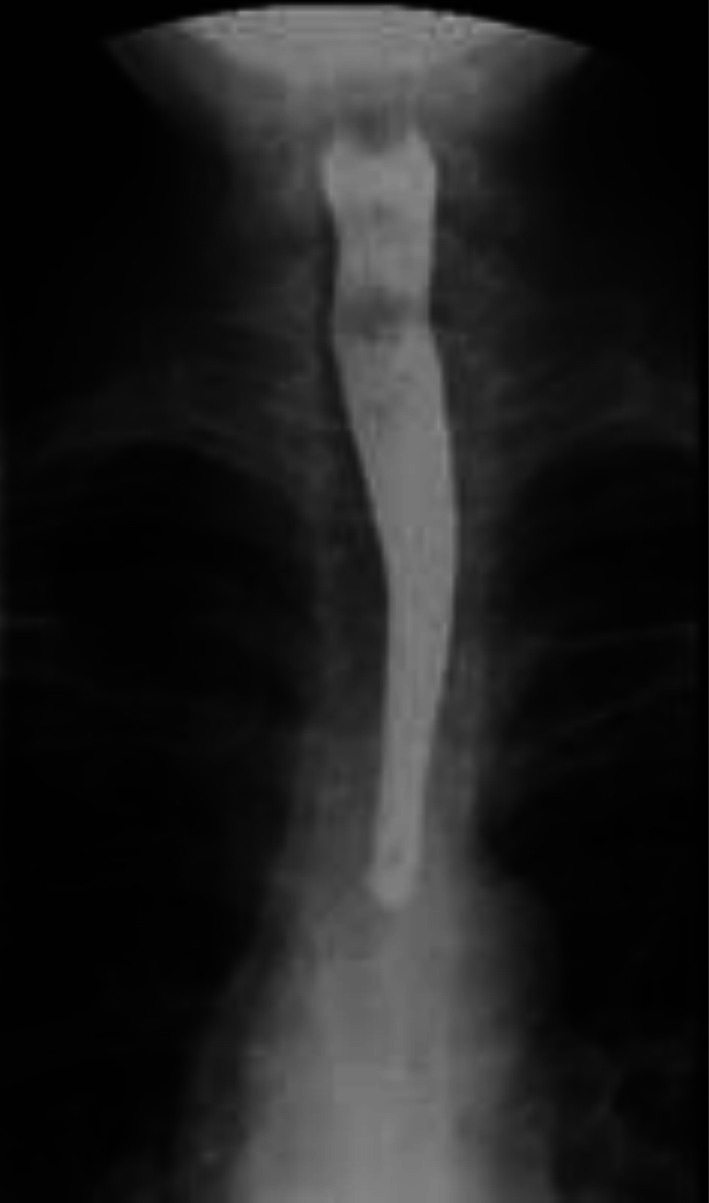
Barium swallow study shows normal esophageal anatomy with no abnormalities. This study effectively excluded Zenker's diverticulum and other esophageal pathologies from the differential diagnosis.

Contrast‐enhanced CT of the neck and chest with 3D reconstruction demonstrated a 4 × 5 cm air‐filled outpouching from the right posterolateral tracheal wall at the level of the 5–7 tracheal rings (Figures 1, 2, 3, 4, 5). By this contrast enhanced CT with 1 mm slice thickness, we assessed the diverticulum's spatial relationship to adjacent neurovascular structures by multiplanar reconstructions (coronal and sagittal) and 3D volume. It optimizes our surgical planning.

**FIGURE 6:**
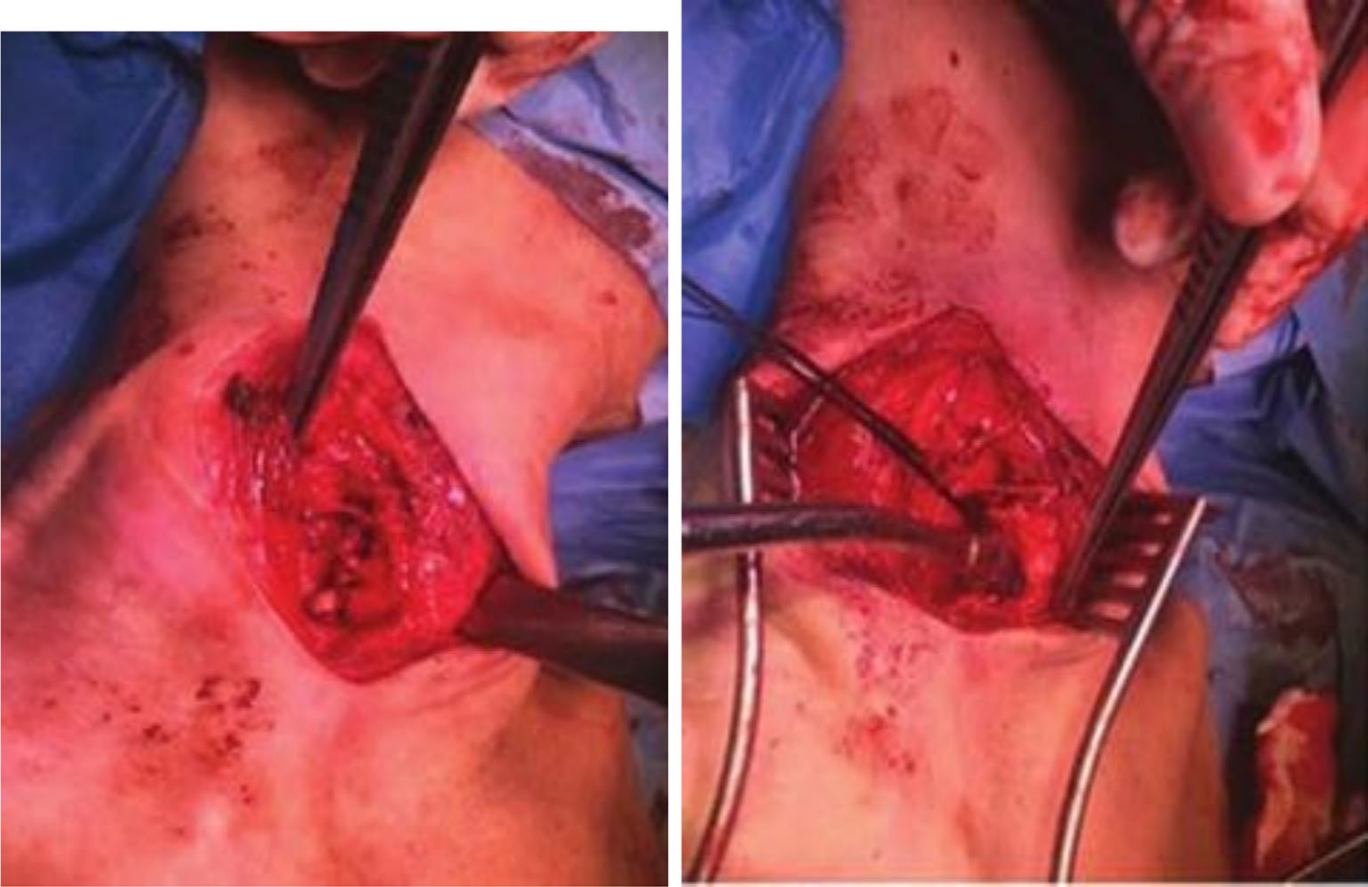
It shows the surgical field after complete excision of the tracheal diverticulum. The tracheal defect is visible and ready for primary repair.

The narrow communication with the tracheal lumen was about 8 mm. There was no evidence of infection or other abnormalities. Barium swallow study excluded esophageal pathology (Figure 6). Flexible bronchoscopy revealed limited mobility of the right vocal cord and clearly identified the diverticulum's ostium in the upper trachea. It confirmed the diagnosis.

**FIGURE 7:**
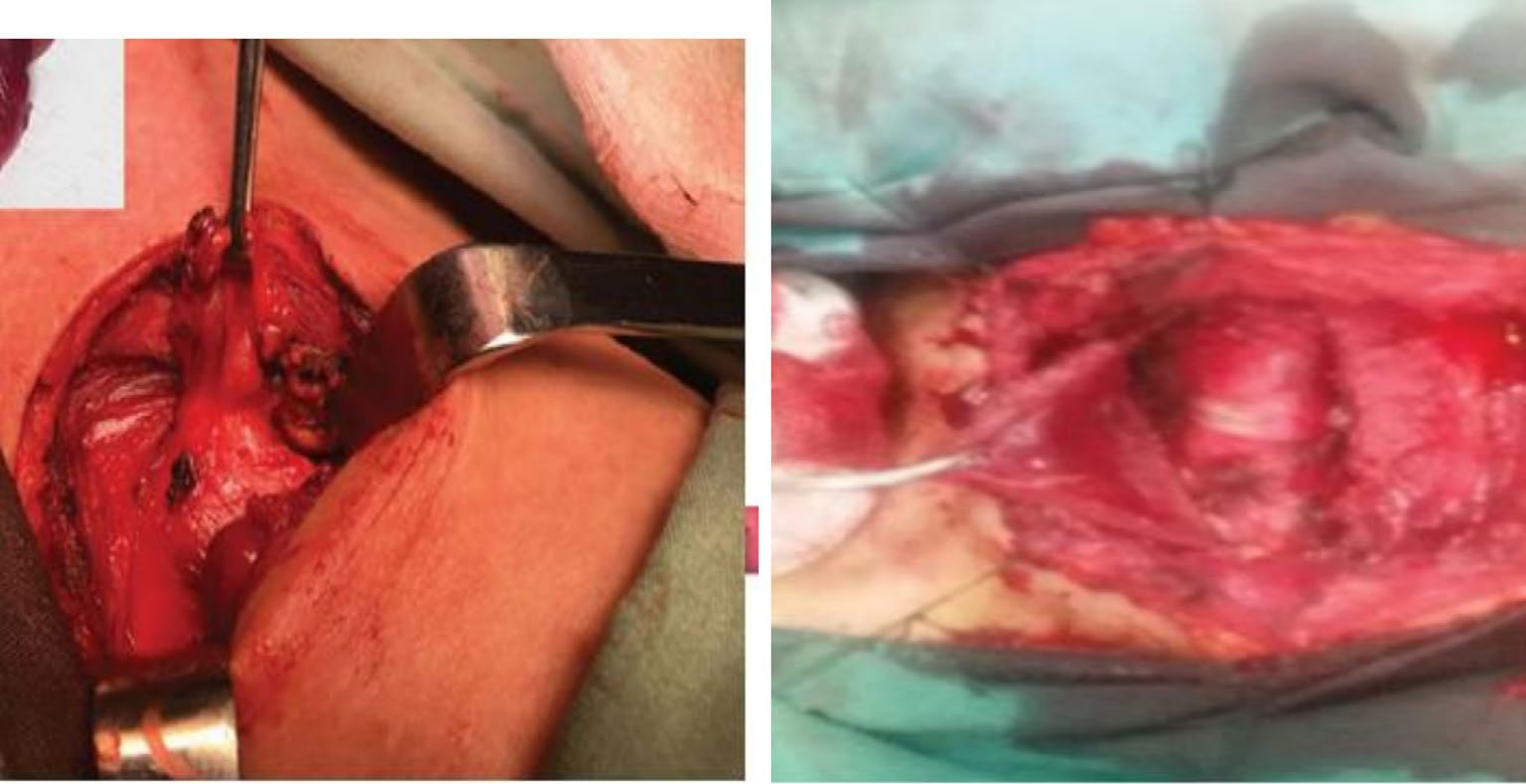
It shows primary closure of the tracheal defect. We used interrupted 2–0 Prolene sutures. The repair was performed with a special technique to ensure airway integrity and prevent complications.

**FIGURE 8:**
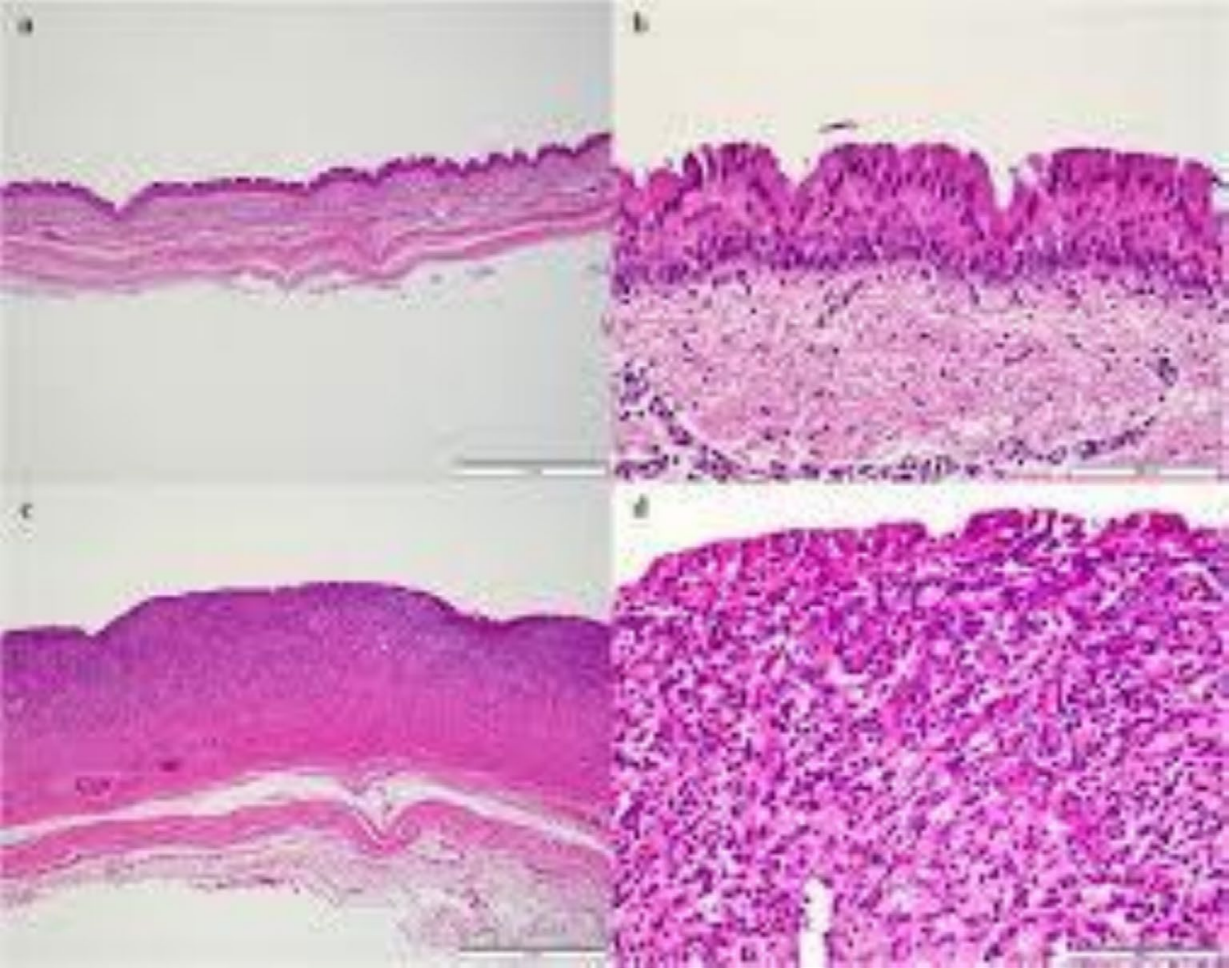
Hematoxylin and Eosin stain of the excised specimen shows a cystic mass that is lined by respiratory epithelium. Lack of cartilage in the wall is characteristic of acquired tracheal diverticulum.

**FIGURE 9:**
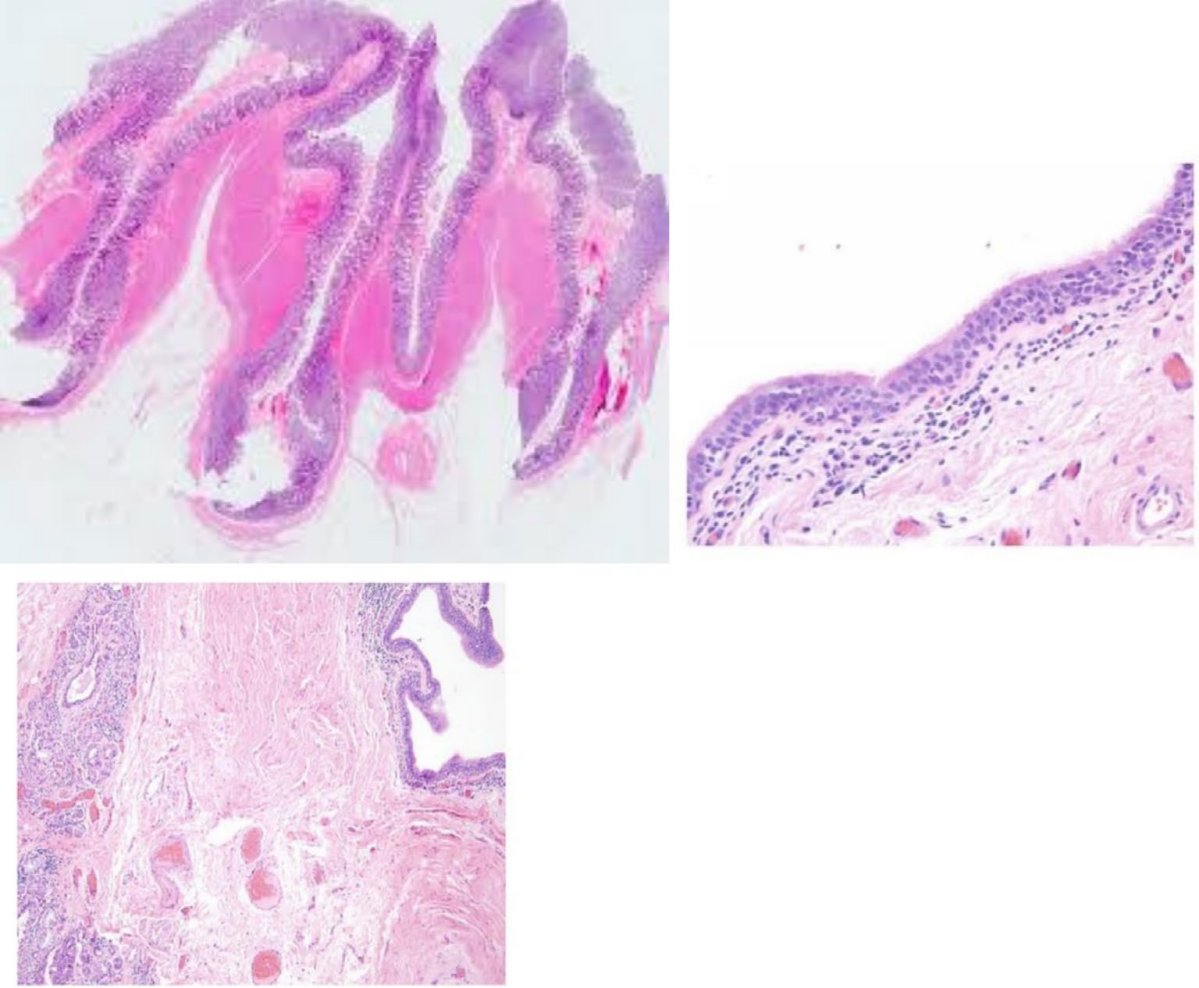
Hematoxylin and Eosin stain demonstrates respiratory‐type ciliated columnar epithelium that is lining the cyst wall with mild chronic inflammatory infiltrate in the surrounding tissue. The absence of cartilage confirmed the diagnosis of acquired tracheal diverticulum.

**FIGURE 10:**
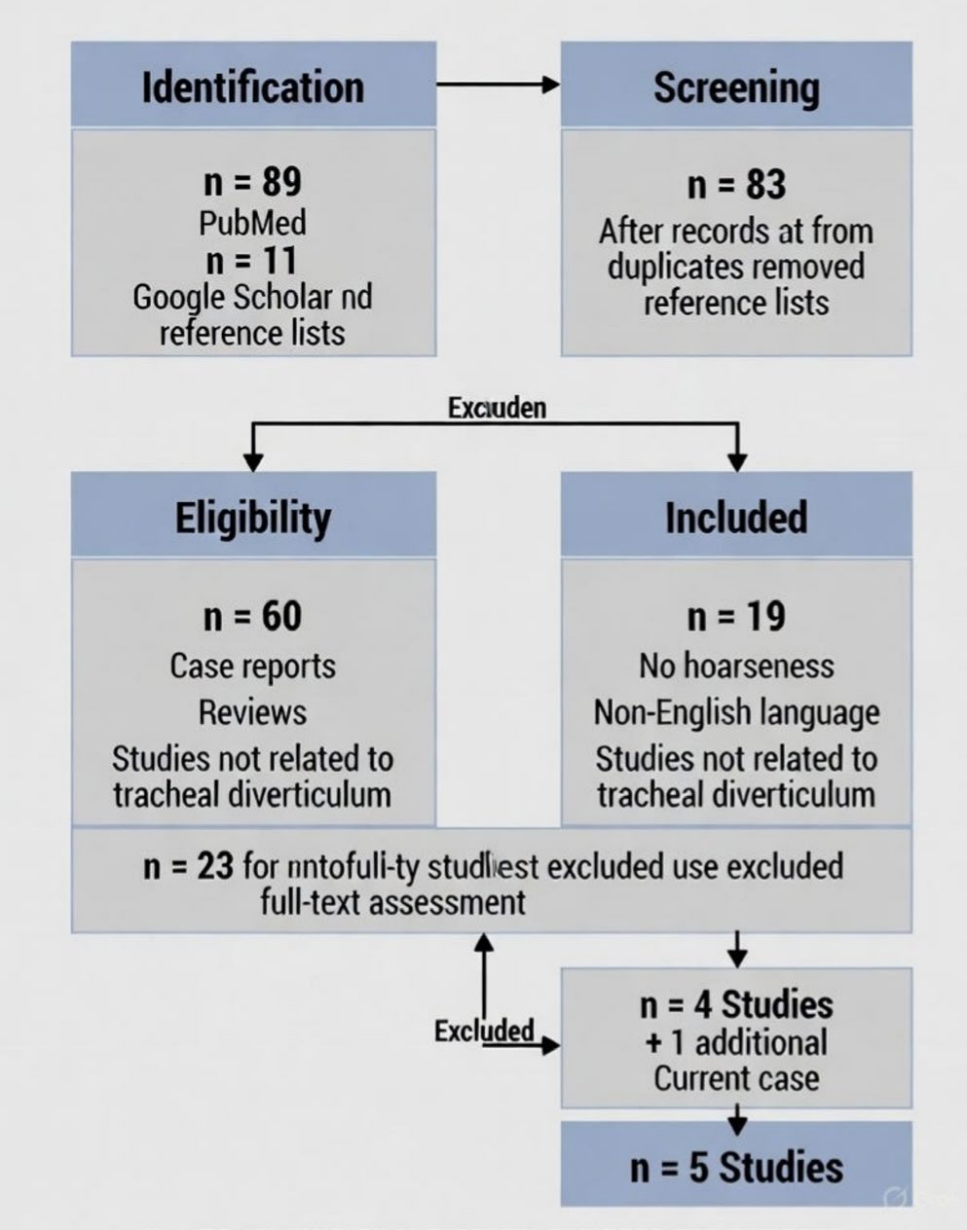
PRISMA Flowchart for Literature Search. It shows the systematic literature search process for tracheal diverticulum cases that present with hoarseness. Initial database searching identified 91 records. After removing duplicates and screening, 23 full‐text articles were assessed. Four cases specifically reported hoarseness with recurrent laryngeal nerve involvement. They have been included in comparative analysis along with 11 additional articles that provide context on epidemiology, diagnosis, and treatment.

Based on the symptomatic presentation and vocal cord dysfunction, we decided to perform surgical excision. It was performed through a right lateral cervical incision under general anesthesia. After careful dissection, it revealed that the right RLN is stretched over the diverticulum. It can explain the vocal cord paresis. The nerve was carefully preserved during the complete excision of the diverticulum. The tracheal defect was repaired primarily with interrupted 2–0 Prolene sutures (Figures 7 and 8). We confirmed repair integrity with positive pressure ventilation under saline. The patient was successfully extubated in the operating room and her postsurgery course went uneventful.

Our pathologists confirmed the cystic spaces, which were lined by respiratory‐type ciliated columnar epithelium and also mild lymphocytic infiltration. It lacked smooth muscle and cartilage (Figure 9). So, it confirmed the acquired TD. At 3‐week follow‐up, the wound had healed completely and hoarseness had resolved. Repeat bronchoscopy demonstrated normal right vocal cord mobility with no residual diverticulum. Her chest CT, 3 months after surgery, revealed complete resolution and normal tracheal anatomy. At 1‐year follow‐up, the patient remained asymptomatic. Her FEV1 also improved to 88% predicted, which can confirm the excellent long‐term outcomes.

## 3 Differential Diagnosis, Investigations, and Treatment

The DDX of paratracheal air‐filled lesions is laryngocele, pharyngocele, Zenker's diverticulum, apical lung herniation, paraseptal blebs, and bronchogenic cysts with tracheal communication [15]. Laryngoceles communicate with the laryngeal ventricle and enlarge with Valsalva. Zenker's diverticulum occurs at the pharyngoesophageal junction with regurgitation and halitosis. Apical lung herniation relates to trauma or increased intrathoracic pressure, while paraseptal blebs are associated with emphysema.

There was a direct tracheal communication. So, it indicated a tracheal diverticulum. We performed a barium swallow to exclude Zenker's diverticulum and it came normal. We performed a chest X‐ray first to exclude obvious pathologies and then proceeded to definitive imaging. Her CT showed no surrounding lung disease, so it ruled out herniation and blebs too. We excluded laryngocele too, because of the infralaryngeal location of the lesion.

Asymptomatic TD require only observation [12], but symptomatic cases need surgery [6]. Our patient had clear indications: large size, extensive symptoms, vocal cord dysfunction, airway obstruction, and complication risk.

We preferred open surgery instead of endoscopy because of the size, nerve involvement, and need for complete excision.

## 4 Literature Review Methods

A comprehensive literature search was conducted to identify all published cases and studies on tracheal diverticula, with particular emphasis on symptomatic presentations and vocal cord involvement. The search was performed across multiple databases including PubMed, Scopus, Web of Science, and Google Scholar. The following keywords and their combinations were used: “tracheal diverticulum,” “tracheal diverticula,” “paratracheal air cysts,” “hoarseness,” “vocal cord dysfunction,” “vocal cord paresis,” “recurrent laryngeal nerve,” “laryngeal nerve compression,” and “symptomatic tracheal diverticulum.”

Articles published between 1990 and 2025 were included in the review to ensure comprehensive coverage of both historical context and contemporary management approaches. The search strategy included case reports, case series, retrospective studies, and original research articles describing clinical presentations, diagnostic approaches, and treatment outcomes of tracheal diverticula. Reference were manually reviewed to identify additional relevant publications (Figure 10).

Inclusion criteria was: (1) TD confirmed by imaging or histopathology; (2) detailing clinical presentations, symptomatic cases; (3) diagnostic modalities and treatment approaches; (4) complications or unusual presentations. Exclusion criteria included: (1) articles not in English; (2) abstracts without full‐text availability; (3) duplicate publications; and (4) other paratracheal pathologies without TD.

The initial search yielded 47 articles from PubMed and 42 from Google Scholar (total *n* = 89). After removing duplicates (*n* = 17) and screening titles and abstracts for relevance (*n* = 72), 26 articles were selected for full‐text review. Of these, 4 case reports specifically documented RLN involvement: Caversaccio et al. [16], Chaudhry et al. [17], Ceulemans et al. [18], and Safarian et al. [19]. These were included in the comparative analysis along with the present case (2025).

Additional relevant literature on TD (*n* = 11) covering epidemiology [1, 2, 3], pathophysiology [5, 7], comprehensive reviews [6], diagnostic approaches [4, 8, 9], treatment methods [10], and complications [13, 14] was included from reference lists of key publications to provide comprehensive context (Table 1).

**TABLE 1:** Published Cases of Tracheal Diverticulum Presenting with Hoarseness and RLN Involvement (1998–2025).

**Case 1**
Study/Year: Caversaccio et al. (1998) Journal: Ann Otol Rhinol Laryngol Country: Switzerland Age/Sex: 56/Male Size: 2.5 cm Location: Right posterolateral Symptom Duration: 6 months Additional Symptoms: Cough, dyspnea RLN Finding: Vocal cord paralysis Diagnostic Methods: CT, Laryngoscopy Surgical Approach: Open cervical excision Voice Recovery Time: 3 weeks Follow‐up Duration: 6 months Complications: None Final Outcome: Complete resolution
**Case 2**
Study/Year: Chaudhry et al. (2014) Journal: Ann Thorac Surg Country: Canada Age/Sex: 54/Female Size: 3 × 4 cm Location: Right posterolateral Symptom Duration: 2 months Additional Symptoms: Cough, dysphagia RLN Finding: Impaired right vocal cord mobility Diagnostic Methods: CT, Bronchoscopy Surgical Approach: Open cervical excision Voice Recovery Time: 2 weeks Follow‐up Duration: 12 months Complications: None Final Outcome: Complete resolution
**Case 3**
Study/Year: Ceulemans et al. (2014) Journal: Ann Thorac Surg Country: Belgium Age/Sex: 38/Female Size: 2 cm Location: Right posterolateral Symptom Duration: 4 months Additional Symptoms: Dyspnea RLN Finding: RLN compression (confirmed intraoperatively) Diagnostic Methods: CT, Laryngoscopy Surgical Approach: Open cervical excision Voice Recovery Time: 3 weeks Follow‐up Duration: Not reported Complications: None Final Outcome: Good recovery
**Case 4**
Study/Year: Safarian et al. (2024) Journal: J Med Case Rep Country: Iran Age/Sex: 62/Male Size: 3.5 cm Location: Posterior Symptom Duration: 8 months Additional Symptoms: Cough, dyspnea, dysphagia RLN Finding: Dysphonia Diagnostic Methods: CT, MRI, Bronchoscopy Surgical Approach: VATS Voice Recovery Time: 4 weeks Follow‐up Duration: 6 months Complications: Minimal pneumothorax (resolved) Final Outcome: Partial improvement
**Case 5 (Current Case)**
Study/Year: Current case (2025) Journal: — Country: Iran Age/Sex: 45/Female Size: 4 × 5 cm Location: Right posterolateral Symptom Duration: 6 months Additional Symptoms: Cough, dysphagia, odynophagia, dyspnea, hemoptysis, choking, neck pain RLN Finding: Nerve stretched over diverticulum (confirmed intraoperatively) Diagnostic Methods: CT with 3D reconstruction, Bronchoscopy, Spirometry (FEV1 78%) Surgical Approach: Open cervical excision with nerve preservation Voice Recovery Time: 3 weeks Follow‐up Duration: 12 months Complications: None Final Outcome: Complete resolution + PFT improvement (FEV1 78% → 88%)

From the 5 identified cases (4 from literature + current case), the following data were systematically extracted: patient demographics (age, sex), diverticulum characteristics (size, location, anatomical position), clinical presentation (specific symptoms, duration), diagnostic methods (imaging, endoscopic findings), laryngeal examination, intraoperative findings regarding RLN, treatment approach (surgical technique), complications, recovery timeline, follow‐up duration, and long‐term outcomes (Table 2).

**TABLE 2:** Comparative Analysis of clinical, anatomical, and outcome characteristics in tracheal diverticulum cases with hoarseness (*n* = 5, 1998–2025).

Characteristic	*n*/*N*	Percentage	Mean ± SD or range	Supporting data/Details
**Demographics**				
Female sex	3/5	60%	—	Chaudhry 2014, Ceulemans 2014, Current 2025
Male sex	2/5	40%	—	Caversaccio 1998, Safarian 2024
Age (years)	—	—	51.0 ± 9.8	Range: 38–62 years
Age > 50 years	3/5	60%	—	Older age predominance
**Anatomical features**				
Right‐sided location	4/5	80%	—	All except Safarian 2024 (posterior)
Posterolateral position	4/5	80%	—	Classic location for acquired TD
Posterior position	1/5	20%	—	Safarian 2024 only
Mean maximum diameter (cm)	—	—	3.0 ± 1.1	Range: 2.0–5.0 cm
Size > 3 cm	3/5	60%	—	Chaudhry, Safarian, Current case
Largest reported size	1/5	20%	4 × 5 cm	Current case (2025)
**Clinical presentation**				
Hoarseness/dysphonia	5/5	100%	—	Defining inclusion criterion
Chronic cough	4/5	80%	—	Most common associated symptom
Dyspnea	4/5	80%	—	Second most common symptom
Dysphagia	3/5	60%	—	Compression of esophagus
Odynophagia	1/5	20%	—	Current case only
Hemoptysis	1/5	20%	—	Current case only
Choking episodes	1/5	20%	—	Current case only
Neck pain	1/5	20%	—	Current case only
Multiple symptoms (≥ 3)	4/5	80%	—	All except Ceulemans 2014
Most extensive symptoms (≥ 8)	1/5	20%	—	Current case (8 symptoms)
Symptom duration (months)	—	—	5.2 ± 2.5	Range: 2–8 months
Duration > 6 months	2/5	40%	—	Caversaccio 1998, Current 2025
**Laryngeal findings**				
Vocal cord dysfunction confirmed	5/5	100%	—	All cases by laryngoscopy/bronchoscopy
RLN compression/involvement	5/5	100%	—	Clinically diagnosed or intraoperatively confirmed
Intraoperative RLN visualization	2/5	40%	—	Ceulemans 2014, Current 2025
Nerve stretching documented	1/5	20%	—	Current case only (novel finding)
Complete vocal cord paralysis	1/5	20%	—	Caversaccio 1998
Partial vocal cord dysfunction	4/5	80%	—	All others
**Diagnostic methods**				
CT scan performed	5/5	100%	—	Gold standard imaging
CT with 3D reconstruction	1/5	20%	—	Current case only
MRI performed	1/5	20%	—	Safarian 2024
Laryngoscopy/Bronchoscopy	5/5	100%	—	Essential for vocal cord assessment
Preoperative spirometry	1/5	20%	—	Current case only (FEV1 78%)
Barium swallow	1/5	20%	—	Current case (to exclude Zenker's)
**Treatment approach**				
Surgical excision performed	5/5	100%	—	All cases treated surgically
Open cervical approach	4/5	80%	—	Preferred method for nerve preservation
VATS approach	1/5	20%	—	Safarian 2024
Complete excision achieved	5/5	100%	—	No partial resections
Nerve preservation documented	2/5	40%	—	Ceulemans 2014, Current 2025
Primary tracheal repair	5/5	100%	—	All cases
**Outcomes**				
Voice improvement achieved	5/5	100%	—	All showed improvement
Complete voice recovery	4/5	80%	—	All except Safarian 2024
Partial voice recovery	1/5	20%	—	Safarian 2024
Mean recovery time (weeks)	—	—	3.0 ± 0.8	Range: 2–4 weeks
Recovery < 3 weeks	2/5	40%	—	Chaudhry 2014, Current 2025
Perioperative complications	1/5	20%	—	Safarian 2024 (minimal pneumothorax)
Major complications	0/5	0%	—	None reported
Recurrence	0/4	0%	—	No recurrence in cases with follow‐up
Follow‐up ≥ 6 months	4/5	80%	—	Adequate long‐term assessment
Follow‐up ≥ 12 months	2/5	40%	—	Chaudhry 2014, Current 2025
Objective functional improvement	1/5	20%	—	Current case (PFT: FEV1 + 10%)
Complete symptom resolution	4/5	80%	—	All except Safarian 2024
**Documentation quality**				
Comprehensive preoperative workup	1/5	20%	—	Current case (CT 3D, spirometry, bronch)
Detailed intraoperative description	2/5	40%	—	Ceulemans 2014, Current 2025
Serial postoperative assessments	1/5	20%	—	Current case (3 weeks, 3 months, 1 year)
Quantitative outcome measures	1/5	20%	—	Current case (spirometry data)

Abbreviations: CT, computed tomography; FEV1, forced expiratory volume in 1 s; MRI, magnetic resonance imaging; PFT, pulmonary function test; RLN, recurrent laryngeal nerve; TD, tracheal diverticulum; VATS, video‐assisted thoracoscopic surgery.

Special attention was given to cases reporting hoarseness or RLN involvement. Descriptive statistics were calculated for continuous variables (mean ± standard deviation) and categorical variables (frequencies, percentages). The present case was systematically compared with previously reported cases to identify common patterns, unique features, and gaps in existing knowledge (Table 3).

**TABLE 3:** Novel contributions of current case (2025) compared to published literature (1998–2024).

Feature category	Literature cases (*n* = 4) 1998–2024	Current case (2025)	Clinical significance
Preoperative assessment			
CT with 3D reconstruction	0/4 (0%)	Yes	Enhanced visualization of diverticulum‐trachea relationship; improved surgical planning
Preoperative spirometry	0/4 (0%)	Yes (FEV1 78%, FEV1/FVC 68%)	Quantified baseline airway obstruction; established objective outcome measure
Systematic vocal cord mobility assessment	4/4 (100%)	Yes (limited right vocal cord mobility)	Standard preoperative evaluation confirmed
Barium swallow for differential diagnosis	0/4 (0%)	Yes (normal)	Systematically excluded Zenker's diverticulum
Comprehensive symptom documentation	Limited (1–4 symptoms)	Extensive (8 distinct symptoms)	Most complete symptomatic presentation reported
Intraoperative findings			
Direct RLN visualization	1/4 (Ceulemans: compression noted)	Yes (detailed anatomical description)	Improved understanding of surgical anatomy
Mechanism of nerve involvement	Not described	Nerve stretched over diverticulum	**Novel finding:** First description of stretching mechanism
Anatomical relationship documented	Minimal description	Detailed (nerve draped over lesion)	Provides anatomical basis for vocal cord dysfunction
Nerve preservation technique	Mentioned but not detailed	Detailed description (careful dissection, liberation)	Practical surgical guidance for future cases
Repair technique specification	Basic description	Detailed (interrupted 2–0 Prolene, transverse orientation)	Technical details for stenosis prevention
Intraoperative integrity testing	Not mentioned	Yes (positive pressure under saline)	Quality assurance measure documented
Symptom complexity			
Number of symptoms	Range: 1–4	8 distinct symptoms	Most extensive presentation
Hemoptysis present	0/4 (0%)	Yes	Rare symptom documented
Choking episodes	0/4 (0%)	Yes	Rare symptom documented
Odynophagia	0/4 (0%)	Yes	Uncommon symptom added to spectrum
Neck pain	0/4 (0%)	Yes	Local compressive symptom
Postoperative documentation			
Repeat bronchoscopy	Limited/not specified	Yes (3 weeks: normal mobility)	**Objective recovery documentation**
Follow‐up imaging	Limited/not specified	Yes (CT at 3 months: complete resolution)	Radiological confirmation of cure
Postoperative spirometry	0/4 (0%)	**Yes (FEV1 88% at 1 year: +10% improvement)**	**Novel: Quantified functional benefit**
Multiple time‐point assessment	1–2 time points	3 time points (3 weeks, 3 months, 12 months)	Comprehensive outcome tracking
Serial vocal cord assessment	Limited	Complete (pre‐op, 3 weeks, documented recovery)	Objective timeline of neural recovery
Outcome measures			
Subjective symptom improvement	4/4 (100%)	Yes (all 8 symptoms resolved)	Complete symptomatic cure
Objective vocal cord recovery	4/4 (100%) clinical assessment	Yes (bronchoscopy‐confirmed normal mobility)	Gold standard confirmation
**Quantified pulmonary function**	**0/4 (0%)**	**Yes (FEV1: 78% → 88%, Δ + 10%)**	**First case with objective PFT improvement data**
Quality of life assessment	0/4 (0%)	Implicit (complete symptom resolution)	Functional improvement documented
Long‐term follow‐up (≥ 12 months)	1/4 (Chaudhry: 12 months)	Yes (12 months, sustained improvement)	Durable outcome confirmed
Unique contributions			
Largest diverticulum with hoarseness	Previous max: 3.5 cm	4 × 5 cm (5 cm maximum diameter)	Largest symptomatic lesion reported
Intraoperative nerve stretching	Never described	**First documentation**	**Novel mechanism elucidated**
Objective airway improvement	Never quantified	**FEV1 + 10% at 1 year**	**First quantitative functional data**
Comprehensive multitimepoint follow‐up	Minimal	Complete (3 stages over 1 year)	Establishes outcome timeline
Combined clinical + functional outcomes	Clinical outcomes only	Clinical + objective PFT data	Most complete outcome assessment

*Note:* The bolded items in Table 3 highlight several key methodological and clinical advancements introduced by our case compared to earlier published literature. Specifically, our case is the first to document the mechanism of recurrent laryngeal nerve injury as stretching rather than compression, offering a pathophysiological basis for the reversibility of hoarseness following surgical intervention. We also report the largest symptomatic diverticulum documented to date, measuring 4 by 5 centimeters, which reinforces the association between diverticulum size and symptom burden. Furthermore, our study introduces objective functional outcome measures, including preoperative and postoperative spirometry demonstrating a 10% improvement in FEV1, a quantitative finding not previously reported. The comprehensive use of 3D CT reconstruction, systematic barium swallow evaluation, and structured multi timepoint follow up establishes a new standard for thorough assessment and longitudinal monitoring in the management of tracheal diverticulum.

## 5 Conclusion

TD should be considered in chronic respiratory symptoms and hoarseness. This condition needs careful surgical intervention. CT can confirm the diagnosis, but bronchoscopy is essential as it can assess the vocal cord function and guide the surgical approach.

Symptomatic TD causes vocal cord dysfunction and requires surgical intervention. Successful outcomes depend on nerve preservation. Our patient's symptom fully resolved, and her vocal cord function became normal. Her spirometry became normal at 1 year. It confirmed the effectiveness of this approach.

This case report and literature review among 27 years (1998–2025) shows that hoarseness is a rare but significant presentation of TD. It can occur in 1.3% of reported symptomatic cases and indicates the RLN stretching. Analysis of all 5 documented cases reveals consistent patterns: female predominance (60%), right posterolateral location (80%), larger diverticulum size (mean 3.0 cm, range 2–5 cm), and favorable surgical outcomes (80% complete voice recovery in 3 weeks, 0% recurrence, 0% major complications). Our case gives several novel points to this limited information: we have first documented the mechanism of nerve stretching. Also, we have the first quantitative data via spirometry (FEV1 78% → 88%, +10%). Our pre surgical assessment was the most comprehensive method (3D CT reconstruction, spirometry), and our lesion was the largest reported diverticulum (4 × 5 cm).

## 6 Discussion

This case presents a woman with hoarseness. The final diagnosis was acquired TD. Our findings were confirmed by surgery. It provides valuable insights into the pathophysiology, diagnostic approach, and surgical management of this uncommon condition.

The anatomical features were matched with documented patterns. Its location and its size are consistent with typical acquired TD [1, 3, 5]. Histopathology showed respiratory epithelium without cartilage or smooth muscle. It distinguishes it from congenital diverticula and tracheogenic cysts [4, 5].

Our systematic literature search identified 4 previously published similar cases from 1998 to 2025 (Table 1). Including the present case (2025), that represents 1.3% of all published TD cases with detailed symptoms [8]. This uncommon condition highlights the clinical significance of hoarseness as a potential indicator of RLN stretching in TD.

Analysis of these 5 cases reveals consistent patterns (Table 2). All vocal cord dysfunctions were confirmed by laryngoscopy or bronchoscopy. Symptom duration was from 2 to 8 months (mean 5.2 ± 2.5 months). Female had predominance (60%, 3/5 cases), while sex distribution is equal in acquired forms [1, 3]. The mean age of 51.0 ± 9.8 years (range 38–62) is consistent with the typical presentation.

The single case with posterior (rather than posterolateral) location [19], represents an anatomical variant.

The mean diverticulum size in symptomatic cases was 3.0 ± 1.1 cm (2.0–5.0 cm). It is larger than typical asymptomatic diverticula, which are often smaller than 2 cm [2, 8]. This correlation suggests that larger diverticula compress more often the RLN (Table 4). Our case presented the largest reported diverticulum (4 × 5 cm, maximum diameter 5 cm).

**TABLE 4:** Size–symptom correlation.

Diverticulum size vs. Number of symptoms		
Study/Case	Diverticulum size (cm)	Number of symptoms
Caversaccio	2.0 cm	1 symptom
Ceulemans	3.0 cm	2 symptoms
Safarian	3.5 cm	4 symptoms
Chaudhry	4.5 cm	3 symptoms
Current Case	5.0 cm	8 symptoms

Previous reports described nerve “compression” [18], “paralysis” [16], or “impaired mobility” [17]. Our observation during the surgery confirmed stretching of RLN, rather than compression. This finding has important implications. It also explains why complete recovery is achievable with careful operation (Tables 5 and 6).

**TABLE 5:** Voice recovery status at final follow‐up (*n* = 5).

Recovery status	Number of cases	Percentage
Complete recovery	4	**80%**
Partial recovery	1	**20%**

*Note:* The bolded value in Table 5, indicating an 80% rate of complete voice recovery, underscores the high efficacy of surgical excision with nerve preservation in patients presenting with hoarseness due to tracheal diverticulum. This result confirms that recurrent laryngeal nerve dysfunction caused by diverticular stretching is often reversible with timely and precise surgical intervention. The single case of partial recovery, representing 20% of the cohort, was associated with atypical clinical features such as a posterior diverticulum location, a VATS surgical approach, older patient age, and longer symptom duration. These factors highlight important clinical variables that may influence functional outcomes and merit consideration in surgical planning.

**TABLE 6:** Recovery timeline—time to voice recovery (*n* = 5).

Recovery time	Number of cases	Notes
2 weeks	1 case	Early recovery
3 weeks	3 cases	Most patients
4 weeks	1 case	Delayed but complete
> 4 weeks	0 cases	—

*Note:* Mean recovery time: 3.0 ± 0.8 weeks.

The pathology revealed herniation of the membranous tracheal wall through structural weakness. It chronically increased the intraluminal pressure [5, 6, 7]. Our patient had a mild obstructive pattern (FEV1 78% predicted) in spirometry. It improved to 88% predicted at 1 year.

The clinical presentation was more extensive than typically reported [5, 8]. Our patient manifested 8 distinct symptoms: chronic cough, dysphagia, odynophagia, dyspnea, hoarseness, hemoptysis, choking episodes, and neck pain. Hoarseness is an uncommon but clinically significant manifestation [5, 6]. Similar nerve compression was documented in other mediastinal masses [20].

In the operation we found that the right RLN is stretched over the diverticulum. Three weeks after surgery hoarseness resolved. It confirms a reversible rather than permanent injury [1].

Our diagnostic approach was the best practice. The x‐ray was unremarkable, but CT provided a diagnosis [3, 4, 9]. By bronchoscopy, we gained complementary information because it helps visualization of the diverticular opening and assessing vocal cord mobility [10].

All 5 documented cases underwent surgery. 80% (4/5) of them underwent an open cervical approach and 1 of them (20%) video‐assisted thoracoscopic surgery (VATS) [19] (Tables 1 and 2). This similar decision among different countries (Switzerland, Canada, Belgium, Iran) reflects an international approach for symptomatic diverticula (Table 7).

**TABLE 7:** Surgical approach distribution.

Surgical approaches used (*n* = 5)		
Surgical approach	Number of cases	Percentage
Open cervical approach	4	**80%**
VATS approach	1	**20%**

*Note:* The bolded data in Table 7, showing that an open cervical approach was used in 80% of cases, reflects the dominant surgical preference for managing symptomatic tracheal diverticula with recurrent laryngeal nerve involvement. This approach is favored because it provides direct visualization, facilitates safe nerve dissection, and allows for complete excision of the diverticulum, all of which are critical for achieving optimal vocal recovery. The single case where a VATS approach was employed, resulting in only partial improvement, suggests that while minimally invasive techniques are feasible, the open cervical method remains the gold standard when nerve preservation is a primary objective.

The preference of open cervical approaches (80%) highlights the importance of direct visualization and dissection in RLN involvement. However, via VATS it could be problematic.

Eighty percent of cases (4/5) achieved complete recovery within 2–4 weeks (mean 3.0 ± 0.8 weeks). The single case [19] had several features of less favorable outcome: the patient age (62 years, which was the oldest in the series), longest symptom duration (8 months), posterior rather than posterolateral location (less typical anatomy), and VATS approach (that could limit direct visualization).

Older patients have lesser neural regenerative capacity. Prolonged duration of nerve injury (> 6 months) causes irreversible axonal damage or fibrosis. The posterior location may involve different anatomical relationships between the diverticulum and RLN. So, VATS has less benefit for identifying and preserving the nerve, although it has advantages like lesser surgical complications. So, early surgical intervention (within 6 months of symptom onset) is important. Open cervical approach is preferred.

There were no major perioperative complications in any of these cases across 27 years. During follow‐up periods (from 6 months to 12 months in the 4 cases), no recurrence was detected. It confirms a 100% cure rate. It was a mild pneumothorax in the VATS case [19], which resolved without intervention.

To rule out the DDXs we need systematic evaluation [11, 12]. Zenker's diverticulum presents with dysphagia and regurgitation [12]. A normal barium swallow effectively excluded that. Laryngoceles located in the supraglottic. So, it has been excluded too. We ruled out the apical lung herniation and paraseptal blebs too because there was no history of lung parenchymal disease [11].

She had the indications of surgery: the symptomatic presentation, large size (4 × 5 cm), vocal cord dysfunction, airway obstruction, and complication risk [2, 6, 9]. Although Bae et al. [2] chose conservative management for asymptomatic cases, Tanrivermis Sayit et al. [6] recommend surgery for symptomatic diverticula. Potential complications include difficult intubation and pneumomediastinum [13] and serious infections [14].

By surgery we did a complete excision and nerve preservation [5, 6]. By right lateral cervical incision, we provided an excellent condition to dissect the nerve carefully. Primary closure was with interrupted 2–0 Prolene sutures in transverse orientation. It decreased the stenosis risk [5]. During the surgery we confirmed the repair integrity by saline.

Considering the size and nerve involvement we chose open cervical approach over endoscopic techniques [9, 10]. Aghajanzadeh et al. [9] noted that VATS is increasingly recommended, because there are surgical difficulties for cysts greater than 10 cm or those, that are adjacent to vital structures. They sometimes need open thoracotomy. The excellent outcome validates our approach (Table 8).

**TABLE 8:** Comparative outcomes—current case vs. literature.

Category	Feature	Literature (*n* = 4)	Current Case	Notes
Preoperative assessment	3D CT reconstruction	0%	100%	✓
	Preoperative spirometry	0%	100%	✓
	Vocal cord assessment	100%	100%	✓
Intraoperative documentation	RLN visualization	25%	100%	✓
	Nerve stretching documented	0%	100%	✓✓
	Nerve preservation technique	Limited	Yes	✓
Postoperative follow‐up	Repeat bronchoscopy	Limited	Yes	✓
	Follow‐up CT imaging	Limited	Yes	✓
	Postoperative spirometry	0%	100%	✓✓
	Multiple time‐points (≥ 3)	25%	100%	✓
Outcome measures	Subjective improvement	100%	100%	✓
	Objective vocal cord recovery	100%	100%	✓
	Quantified PFT improvement	0%	100%	✓✓
	Complete symptom resolution	75%	100%	✓

Patient was discharged on day 3. Her hoarseness resolved at 3 weeks, and on repeat bronchoscopy, we saw a normal vocal cord mobility. At 3 months the radiological findings were resolved completely, and her pulmonary function test was improved at 1 year too. It confirms the effectiveness of the surgery and the capacity of neural recovery.

This several points deserve particular attention [5, 6, 9]. The detailed documentation of vocal cord dysfunction provides objective evidence of nerve involvement and recovery. Pulmonary function testing offers quantitative data on functional improvement. Direct visualization of nerve stretching during the surgery confirms the anatomical basis for hoarseness.

There have been limitations. As a single case, generalizability is limited. One‐year follow‐up, although demonstrated excellent outcomes, lacks long‐term recurrence data. Aghajanzadeh et al. [9] reported median 70‐month follow‐up with no recurrence in 68 mediastinal cysts. It suggests long‐lasting results. It also lacks a formal quality‐of‐life assessment and a comprehensive pulmonary function test before surgery [8, 9].

Future research should include prospective multicenter studies with standardized protocols. They should investigate the nerve involvement predictors and compare the open versus minimally invasive approaches [9, 10], and include long‐term follow‐up to examine the recurrence and quality of life [3, 6].

Advanced 3D reconstruction and patient specific modeling may improve our visualization of anatomical relationships in tracheal diverticula. It helps us to make better surgical planning. Recent studies confirm this as the best approach in thoracic surgery [9]. Especially dynamic imaging protocols can capture airway–mediastinal interactions during respiration [21, 22]. Future studies should investigate the virtual surgical simulation and quantitative functional analysis. This improves patient selection and prediction of recovery in rare presentations with recurrent laryngeal nerve involvement.

In overall, symptomatic acquired tracheal diverticula with hoarseness and vocal cord dysfunction require surgical intervention [5, 6]. Preoperative evaluation with CT and bronchoscopy [3, 9, 10], an accurate surgical technique with nerve preservation, and systematic follow‐up can resolve the symptoms completely and result in functional improvement. With proper treatment, patients experience full symptom resolution and restoration of normal function [8, 9].

## Author Contributions

**Zahra Sadin:** conceptualization, data curation, formal analysis, funding acquisition, investigation, methodology, project administration, resources, software, supervision, validation, visualization, writing – original draft, writing – review and editing. **Manouchehr Aghajanzadeh:** conceptualization, data curation, formal analysis, funding acquisition, investigation, methodology, project administration, resources, software, supervision, validation, visualization, writing – original draft, writing – review and editing. **Mohammadreza Sadin:** conceptualization, data curation, formal analysis, funding acquisition, investigation, methodology, project administration, resources, software, supervision, validation, visualization, writing – original draft, writing – review and editing.

## Funding

The authors have nothing to report.

## Ethics Statement

The authors have nothing to report.

## Consent

Written informed consent was obtained from the patient for publication of this case report and accompanying images.

## Data Availability

All data generated or analyzed during this study are included in this published article.
